# Strengthening Integrated Approaches for Family Planning and Menstrual Health

**DOI:** 10.9745/GHSP-D-23-00080

**Published:** 2023-10-30

**Authors:** Emily Hoppes, Kate H. Rademacher, Lucy Wilson, Tanya Dargan Mahajan, Katrina Wilson, Marni Sommer, Marsden Solomon, Eva Lathrop

**Affiliations:** a FHI 360, Durham, NC, USA.; b Independent consultant, Chapel Hill, NC, USA.; c Rising Outcomes, Hillsborough, NC, USA.; d The Pad Project, New Delhi, India.; e MSI Reproductive Choices, London, United Kingdom.; f Department of Sociomedical Sciences, Mailman School of Public Health, Columbia University, NY, USA.; g FHI 360, Nairobi, Kenya.; h Population Services International, Washington, DC, USA.

## Abstract

FP and menstrual health integration has the potential to improve individuals' health and well-being. The authors describe potential ways to integrate FP and menstrual health, outlining steps that stakeholders can take in designing integrated approaches.

## Key Messages

- The family planning (FP) and menstrual health (MH) fields share goals and areas of work, serve similar populations, and have the potential to learn from and enhance one another, but they are not effectively integrated, which can result in missed opportunities to improve individuals' health, well-being, and dignity.
- Integrating FP and MH ensures that both FP and MH commodities and services are provided under a single programmatic umbrella that may include both same-day, co-located services and referral-based approaches.
- Potential ways to integrate FP and MH include (1) improving education and awareness; (2) integrating delivery of FP and MH commodities and services within health systems; (3) improving integrated counseling on FP and MH; (4) including evidence-based methods that rely on menstrual tracking in FP method provision; (5) addressing the issue of menstrual status as a barrier to FP; (6) including contraceptives as an option for managing menstrual disorders and pain; (7) implementing social and behavior change communication and advocacy for stakeholder engagement; and (8) strengthening national policies and guidelines.

## BACKGROUND

Family planning (FP) and menstrual health (MH) are closely related fields that are often not effectively integrated, which can result in missed opportunities to improve the health, well-being, and dignity of individuals. Many actors in the field have long touted MH education and programs as a key entry point for broader reproductive health efforts, especially among adolescents.^1^^,^^2^ Likewise, those working in FP recognize the impact of contraceptives on menstruation and the need for counseling and education to address this issue.^3^ Recent work has brought together experts from the fields of both FP and MH.^3^^,^^4^ In breaking down silos between these 2 fields, a growing interest in the topic of FP-MH integration has emerged. Experts agree that greater efforts should be made to proactively link FP and MH policies and programs, including through provider training and capacity-strengthening, community and school-based education and outreach, service provision, and program evaluation. In this commentary, we propose a definition of FP-MH integration, summarize the rationale for this approach, describe potential areas for expanded FP-MH integration, and outline steps that key stakeholders—including governments, donors, program managers, and health care providers—can take in the design of integrated approaches.

### Rationale for FP-MH Integration

Many linkages exist between FP and MH, but they are often underappreciated or ignored, and the fields have, for the most part, remained siloed.^4^ This is unfortunate because the sectors share goals and areas of work, serve similar populations, and have the potential to learn from and enhance each other.^5^ Both FP and MH operate under the larger umbrella of sexual and reproductive health and rights (SRHR), and both emphasize the importance of body literacy, bodily autonomy, choice, and self-care. Both fields have a shared goal to improve the health, well-being, and dignity of women, girls, and other people who menstruate and can face challenges of stigma, misinformation, and navigating complex social and gender norms. They both struggle to reach people across the reproductive life course, with MH sometimes being criticized for focusing too narrowly on adolescents and ignoring the needs of older menstruators, while country-level FP services often focus on those who are post-adolescent or in the middle of life and can struggle to reach adolescents. While this is true for the sectors on a conceptual level, MH is a much younger field that does not have the reach of the more established field of FP. Integrated approaches could help address these challenges and increase the reach and impact of both fields. Insights can be gained from other fields of health integration, including FP-HIV^6^^,^^7^ and FP-immunization^8^ (Box). These insights were considered in the design of the programmatic guidance we describe later and have been used to develop the following formal definition of FP-MH integration:

Integrating FP and menstrual health could help address challenges of stigma, misinformation, and navigating complex social and gender norms and increase the reach and impact of both fields.

BOX Insights From Other Integrated Approaches

- There are several common modalities for integrating services, ranging from same-day, co-located service delivery to referral-based approaches. The chosen modality will depend on the availability of resources, infrastructure, national policies in place, types of facilities and workers available, and other factors.^8^ Because of the wide variety of available approaches, it is key to define when, how, and where in the health system the integration should occur in different contexts.
- When integrating new services into an existing program, it is important to avoid negatively impacting the functioning of those systems. Integration should enhance existing services, not hinder them.^7^ Likewise, it is important to conduct formative research with target populations and solicit input from experts in each field before designing integrated approaches to address contextual factors, such as social and cultural norms.^6^
- Integration efforts should consider the needs and abilities of providers and ensure that they have the time and capacity to offer enhanced services.^6^^–^^8^

The integration of FP and MH ensures that both FP and MF commodities and services are provided under a single programmatic umbrella, which may include both same-day, co-located services and referral-based approaches. “Services” refer to a wide range of programmatic elements, including training, education, counseling, advocacy, social behavior change communication, and access to products, facilities, resources, and care.

## KEY AREAS FOR FP-MH INTEGRATION

In conversations with both MH and FP experts, a number of potential areas for integration have emerged. Although experts agree that each of these suggested areas of integration shows potential, some areas have a more limited evidence base. This is not an exhaustive list but rather a launching point for further conversation and research in developing a more comprehensive approach to FP-MH integration.

### 1. Improve Education and Awareness

Due to gender inequality and harmful gender norms that exist in most spaces, stigma and misinformation can impact both the FP and MH fields and can, in part, be addressed through educational outreach and dissemination of correct and accessible information (Table 1).^9^^–^^16^ This includes using evidence-based curricula and teaching methods, such as comprehensive sexuality education,^9^^,^^17^ to provide information about FP and MH, including information about the menstrual cycle and how it relates to pregnancy and fertility. A growing body of evidence suggests that providing young people with age-appropriate comprehensive sexuality education improves their ability to communicate to family members and health care providers about reproductive health and more easily access services such as FP later in life; this includes an increased ability to negotiate for safer sex and increased control over pregnancy planning and prevention.^18^^,^^19^ Additionally, puberty and sexual health education and training with men and boys, educators and school staff, parents/guardians, community-based workers (across sectors), and community and faith leaders that align with and support the education provided by schools, community health workers, and other channels creates gender-supportive environments for MH management and FP use.^20^^–^^22^

**TABLE 1.** Programmatic Guidance for Family Planning and Menstrual Health Integration on Education and Awareness^a^

Preadolescence and Adolescence	Mid-Life and Reproductive Years	Perimenopause and Menopause
Include evidence-based puberty and comprehensive sexuality education for youth/adolescents that includes age-appropriate information on menstruation and fertility, managing menstrual bleeding and MH, and FP, including CIMCs, across settings, including for both in- and out-of-school youth of all genders. Follow evidence-informed guidance on comprehensive sexuality education.^9^	Continue to provide evidence-based sexuality education and information about menstruation and fertility, managing menstrual bleeding and pain, MH, and FP.	
	**For mid-life and reproductive years:** Counseling and education should include information about postpartum return to fertility.	**For perimenopause and menopause:** Counseling and education should include information about perimenopause and menopause.
Expand access to evidence-based tools that provide access to FP and MH education, products, and services to people of all ages and genders, including tools that can be accessed directly by individuals through digital channels. Examples of existing tools that provide integrated information include AskNivi,^10^ Managing Menstruation: Know Your Options,^11^ Natural Cycles,^12^ and Love Matters.^13^		
Train and strengthen the capacity of CHWs and hold them accountable in providing sensitive, evidence-based counseling and education about menstruation and fertility, managing menstrual bleeding, pain and disorders, MH, and FP, including CIMCs.		
**For preadolescence and adolescence:** Counseling and education should be age-appropriate and youth-responsive.	**For mid-life and reproductive years:** Counseling and education should include information about postpartum return to fertility.	**For perimenopause and menopause:** Counseling and education should include information about perimenopause and menopause.
Provide support information and education to stakeholders, such as educators and school staff, parents/guardians, community-based workers (across sectors), peer educators, and community and faith leaders, that align with and support the education provided by schools, CHWs, and other channels.	Train and strengthen the capacity of stakeholders such as community-based workers (across sectors) and community and faith leaders to provide sensitive, evidence-based education about FP, MH, and CIMCs.	
		**For perimenopause and menopause:** Education should continue throughout perimenopause and until menopause is confirmed.

Abbreviations: CHW, community health worker; CIMC, contraceptive-induced menstrual change; FP, family planning; MH, menstrual health.

a In all areas of integration, conduct programmatic research, implementation science, and routine or enhanced monitoring and evaluation that can be used to inform and improve future programs.

### 2. Integrate Delivery of FP and MH Commodities and Services Within Health Systems

FP and MH can be deliberately integrated by providing services at the same delivery point and by offering referrals (Table 2). This should be grounded in the separate and growing evidence bases that exist for providing FP^23^^,^^24^ and MH^14^^,^^25^ commodities and services and should apply insights from the other areas of health systems integration (HIV-FP and FP-immunization) described earlier. This could include providing all or some of the following under a single program umbrella: MH information, FP counseling, menstrual product provision, FP method provision, and care for menstrual disorders. This level of integration could potentially enhance points of engagement with target groups across the reproductive life course, including often-ignored populations (i.e., perimenopausal women and out-of-school youth), resulting in enhanced service provision and improved SRHR outcomes. It also has the potential to increase cost-effectiveness with the improved efficiency of same-day, co-located service provision. One example of integrated delivery is the CHIEDZA program in Zimbabwe,^26^ which provided menstrual products and FP counseling as a part of a broader package of SRH services. This study found that integration acted as a facilitator to engagement with SRH services overall.^26^ Training and capacity-strengthening with health care providers and anyone else responsible for the delivery of FP and MH services is also important to ensure quality of care.^27^^,^^28^ This could include training on integrated counseling approaches, as we describe in the next area of integration.

**TABLE 2.** Programmatic Guidance for Family Planning and Menstrual Health Integration by Improving Systems-Level Interactions^a^

Preadolescence	Adolescence	Mid-Life and Reproductive Years	Perimenopause and Menopause
	Intesgrate delivery of MH commodities and services into FP within health systems. Provide affordable, high-quality MH products and facilities, including clean, private toilets with space for washing and disposal, and other resources to FP clients during counseling and/or service provision and/or referrals for products and services. Recognize that FP users may need more, less, or different MH products when they are using contraception and that this can change over time.		
	Integrate the delivery of FP commodities and services into MH programs. Provide affordable, high-quality FP services, including counseling and method provision and/or referrals for FP services as part of both school and community-based MH programs.		
	Train and strengthen the capacity of providers and hold them accountable to delivering integrated care. Ensure that those providing sexual and reproductive health services are trained in both comprehensive FP and MH counseling as described in the section above, including training on contraceptive-induced menstrual changes and management of menstrual disorders, as well as youth-responsive services.		
	Promote self-care,^29^ including self-reassurance about contraceptive-induced menstrual changes. Ensure people have the information they need and reliable access to MH products, facilities, and other resources including self-care options for menstrual pain. Support individuals to gain the self-efficacy and bodily autonomy they need to use resources with confidence.		
Ensure FP users are included in MH research and programs, including research involving menstrual products and development of MH standards. Consider the needs and preferences of end users in a holistic way that explicitly includes both FP and MH when designing and implementing MH or FP research, programs, products, systems, and standards.			
Revise health management information systems and reporting tools to support and report on integrated care, including adding information about MH to existing FP registers.			

Abbreviations: FP, family planning; MH, menstrual health.

a In all areas of integration, conduct programmatic research, implementation science, and routine or enhanced monitoring and evaluation that can be used to inform and improve future programs.

### 3. Improve Integrated FP-MH Counseling

FP counseling is an important entry point for providing MH information, including information on the menstrual cycle, menstrual product availability, pain management, and diagnosis and treatment of menstrual disorders. This should include referrals to outside services (Table 2) as well as a focus on self-care, which can enhance reproductive health outcomes.^29^^,^^30^ Providing this basic MH information is also important in addressing the issue of contraceptive-induced menstrual changes (CIMCs). Evidence indicates that CIMCs frequently contribute to discontinuation and nonuse of contraception.^3^^,^^31^^,^^32^ In addition, preliminary evidence suggests that high-quality counseling on CIMCs, including information about noncontraceptive health benefits, can help users make well-informed decisions about the specific method(s) that best meets their needs.^33^ The “NORMAL” job aid, published in 2019, uses a simple mnemonic to counsel users to understand CIMCs, including potential health benefits and lifestyle advantages.^34^^,^^35^ Beyond information provided by tools such as NORMAL, FP counseling should also provide information on the management of CIMCs. In addition, education and conversations about MH and the menstrual cycle, as described in Table 1, are important to ensure that individuals are ready for these counseling conversations later in life. Table 3 provides more details on this area of integration.

**TABLE 3.** Programmatic Guidance for Family Planning and Menstrual Health Integration by Improving Client-Level Interactions Within Health Systems^a^

Preadolescence	Adolescence	Mid-Life and Reproductive Years	Perimenopause and Menopause
Provide information and counseling on MH, including information about the full range of available options for managing menstrual (and contraceptive-induced) bleeding and pain, including information on self-care options and if feasible, access to, or at least information on where to access, commercial menstrual products locally, using tools such as Managing Menstruation: Know Your Options.^11^ Recognize that FP users may need more, less, or different MH products when they are using contraception and that this can change over time.			
Provide access to comprehensive youth-responsive services^30^ that include MH education and information about the full range of MH and FP, including self-care options, to ensure smooth transition into puberty and to ensure that future MH and FP needs are met as soon as they arise. Ensure that services are age-appropriate and welcome all genders.			
	Provide information on FP, including on the full contraceptive method mix including complete and correct information about fertility-based awareness methods and lactation amenorrhea method options.^b^ If the client chooses to use FP, provide effective, evidence-based counseling during and after method selection about potential contraceptive-induced menstrual changes, using provider job aids such as the NORMAL tool.^34^		
	Provide adequate support and clinical treatment for undesirable contraceptive-induced menstrual changes. Ensure adequate follow-up services and counsel FP users that they can return at any time if they have questions or concerns.		
	Provide effective, evidence-based postpartum counseling including products for the management of postpartum bleeding, information about the return of menstruation and fertility after pregnancy and after FP use, as well as support on tapering off FP when trying to conceive.		
	Promote use of the pregnancy checklist^41^ and/or provide access to low-cost pregnancy tests to ensure same-day provision of contraceptive methods among women seeking services when they are not menstruating (i.e., to ensure providers do not rely on the presence of menses as an indicator that a client is not pregnant before providing contraceptive methods.)		
			Provide information and services related to perimenopause and menopause, including how to manage symptoms and counsel on contraceptive use during this life stage. Also, ensure perimenopausal people have access to low-dose contraceptives as an option to relieve menopause symptoms.
Ask about and address concerns about menstruation and menstrual discomfort, including diagnosis and treatment of disorders. Even in the absence of a diagnosis, provide information on management of symptoms, which should include education on self-care (light exercise, stretching and/or yoga, applying heat such as a hot water bottle, taking ibuprofen or naproxen, and other evidence-based self-care options), contraception, and other available options.			

Abbreviations: FP, family planning; MH, menstrual health.

a In all areas of integration, conduct programmatic research, implementation science, and routine or enhanced monitoring and evaluation that can be used to inform and improve future programs.

b Fertility-based awareness methods are not recommended for early adolescents and during perimenopause because menstrual cycles are unpredictable during these times.

FP counseling is an important entry point for providing MH information, including information on the menstrual cycle, menstrual product availability, pain management, and diagnosis and treatment of menstrual disorders.

### 4. Include Evidence-Based Methods That Rely on Menstrual Tracking in FP Method Provision

Fertility awareness-based methods (FABMs) and the lactation amenorrhea method are important FP options, especially for those who prefer nonhormonal approaches and for people seeking postpartum FP, respectively.^36^^,^^37^ FABMs include calendar-based methods that rely on tracking menstrual cycle dates as well as methods that, in addition to menstrual cycle tracking, rely on fertility biomarkers, including cervical mucus, basal body temperature, and metabolites of estradiol and luteinizing hormone in the urine.^38^ Some FABMs are more effective than others, and there are a number of resources and job aids available for these.^36^^,^^38^ An essential aspect of most FABMs is that users learn about their menstrual cycles more comprehensively during counseling as compared to other FP methods. This is because all FABM methods rely on ensuring a couple's understanding of and ability to track the menstrual cycle and identify fertile days. All FABMs are based on partner cooperation and familiarity with the concepts of menstruation and biomarkers of fertility.^39^ The lactation amenorrhea method requires education about the impacts of breastfeeding on the menstrual cycle, which is also enhanced by an understanding of the menstrual cycle more broadly. Table 3 provides more details on this area of integration.

### 5. Address the Issue of Menstrual Status as a Barrier to FP

Providers often rely on the presence of menses as an indicator that someone is not pregnant before providing contraceptive methods; this can create a barrier if someone comes to the clinic on a day they are not menstruating.^40^ In response, a pregnancy checklist tool was developed to address this issue.^41^ It is a job aid with simple questions that a provider can ask a client to rule out pregnancy based on criteria endorsed by the World Health Organization.^42^ Evaluations of the pregnancy checklist have demonstrated that the tool can be effective in ruling out pregnancy for more than 85% of people and use of the job aid can significantly reduce the proportion of clients being turned away due to menstrual status, thus improving access to same-day contraceptive services.^43^^–^^45^ Research also shows that increasing the availability of simple, low-cost pregnancy tests^46^^,^^47^ in FP programs can reduce barriers to FP access,^48^ and a job aid is available to assist providers in deciding when to use the pregnancy checklist versus a pregnancy test.^44^ In addition to these tools, education about the menstrual cycle among both providers and clients could be beneficial in addressing this issue. Table 3 provides more details on this area of integration.

### 6. Include Contraceptives as an Option for Managing Menstrual Disorders and Pain

While not usually a stand-alone treatment, contraceptives can be an important option for those managing menstrual pain and disorders like endometriosis and polycystic ovarian syndrome.^49^^,^^50^ Health care providers should receive information about these options in their training, including being made aware that contraceptives are only a single part of a comprehensive care plan for menstrual disorders. In addition, those managing programs that provide MH education should include this information during educational sessions and integrate referrals to FP providers who have expertise in the management of these disorders. Table 3 and Table 4 provide more details on this area of integration.

**TABLE 4.** Programmatic Guidance for Family Planning and Menstrual Health Integration by Reaching Populations with Special Needs^a,b^

Adolescence	Mid-Life and Reproductive Years	Perimenopause and Menopause
When providing services to populations with special or unique needs,^b^ ensure that both their MH and FP needs are adequately addressed and counsel on contraceptive-induced menstrual changes accordingly. Ensure that individuals are not denied their rights to information about sexual and reproductive health, including MH and FP, and consent to FP method use/provision.		
Ensure populations with special or unique needs are included in FP and MH research and product introduction programs and that these research studies and programs are informed by the populations they are serving and designed to be as accessible as possible.		
Ensure all individuals with menstrual discomfort and/or disorders have adequate counseling and access to contraception as a management or prevention option.		
Ensure people with menstrual disorders are included in FP and MH research and product introduction programs when possible.		

Abbreviations: FP, family planning; MH, menstrual health.

a In all areas of integration, conduct programmatic research, implementation science, and routine or enhanced monitoring and evaluation that can be used to inform and improve future programs.

b Including youth, perimenopausal people, people with disabilities, people living with HIV, postpartum people, refugees, migrants or other mobile populations, sex workers, people in the lesbian, gay, bisexual, transgender, queer community, survivors of abuse and violence, and those who are incarcerated, among others.

### 7. Implement Social and Behavior Change Communication and Advocacy Programs for Stakeholder Engagement

Stakeholder engagement and education are essential to the success of any SRHR program, including for both FP and MH, to create supportive environments at all socio-ecological levels (Table 5). This type of stakeholder engagement can include providing education and information through social and behavior change communication (SBCC) and advocacy among politicians and policymakers.^14^^,^^51^^–^^53^ These types of SBCC and advocacy interventions are essential for building awareness and demand for FP-MH services. Table 5 provides more details on implementing SBCC and advocacy programs.

**TABLE 5.** Programmatic Guidance for Family Planning and Menstrual Health Integration by Engaging Stakeholders and Strengthening National Policies^a^

	Preadolescence	Adolescence	Mid-Life and Reproductive Years	Perimenopause and Menopause
Implement SBCC and Advocacy Programs	Include messaging about FP, MH, and contraceptive-induced menstrual changes in SBCC campaigns and interventions, including interventions that destigmatize and make these topics more understandable to relevant audiences.			
	Advocate at the policy level to ensure decision-makers are educated on the issues of FP, MH, and contraceptive-induced menstrual changes and aware of the best ways to include these issues in policy-level decisions.			
Strengthen National Policies and Guidelines	Review and update FP, MH, and SRHR policies to ensure that adequate, evidence-based information about FP-MH integration and contraceptive-induced menstrual changes is included and promoted.			
	Review and update FP, MH, and SRHR guidelines to ensure that adequate, evidence-based information about FP-MH integration and contraceptive-induced menstrual changes is included. Update clinical guidance and training for health care providers as needed.			

Abbreviations: FP, family planning; MH, menstrual health; SBCC, social and behavior change communication; SRHR, sexual and reproductive health and rights.

a In all areas of integration, conduct programmatic research, implementation science, and routine or enhanced monitoring and evaluation that can be used to inform and improve future programs.

### 8. Strengthen National Policies and Guidelines

Supportive policies and productive collaborations with ministries of health, water and sanitation, gender, and/or education will lead to more successful integrated FP-MH programs. This is because effective policies create enabling environments, remove barriers, and can lead to resource mobilization, all of which improve access and programs.^54^^,^^55^ Several organizations working in SRHR globally have developed technical guidance on the integration of MH into SRHR (including FP) that can be used to inform future policies and guidelines.^56^^–^^58^ Such global guidance documents are important for collaboration with global actors such as the World Health Organization and United Nations Population Fund and to ensure consistency across policies and guidelines. Table 5 provides additional details on strengthening national policies and guidelines.

Supportive policies and productive collaborations with ministries of health, water and sanitation, gender, and/or education will lead to more successful integrated FP-MH programs.

### Programmatic Guidance for FP-MH Integration

Stakeholders—including governments, donors, program managers, and health care providers—can facilitate stronger linkages between FP and MH through integrated models. The table in the Supplement combines all the evidence-based approaches in Tables 1–5 and is meant to guide stakeholders through the process of designing these integrated approaches on many different levels and for individuals across their reproductive life course. As stakeholders begin to design interventions, they should first determine in which areas of integration they will be working (i.e., education, SBCC, service delivery at the client level, etc.) and then determine the population they hope to reach. Suggested activities are provided where these 2 categories intersect. Stakeholders are encouraged to be comprehensive and reach populations with special needs whenever possible.

## CONCLUSION

FP-MH integration at all socio-ecological levels and across the reproductive life course has the potential to significantly improve the health and well-being of women, girls, and other people who menstruate. Program designers and implementers working in SRHR, FP, and MH should consider implementing elements of FP-MH integration into their programs using the evidence-informed guidance provided here. This will require significant cross-sector collaboration between MH and FP, as well as with other related fields such as education and water, sanitation, and hygiene. Because current evidence in this area is limited, there is also a significant need for programmatic research, implementation science, and routine or enhanced monitoring and evaluation that can be used to inform and improve future FP-MH integration programs. Any program that uses the guidance provided here should evaluate its FP-MH programs as they are being integrated and, when appropriate, incorporate research to better understand the relative impact of different integrated approaches. For example, studies are needed to examine how interventions can be designed to improve contraceptive satisfaction and use, how integrated approaches can impact users' menstrual cycle experiences, whether co-located service delivery is more or less effective than referrals, and what type of providers are best positioned to provide integrated services and how they can best be trained to do so. This research should be guided by agendas and research priorities that have been established systematically through expert consultation, community input, and by taking current evidence into account.^3^^,^^58^

## Supplementary Material

GHSP-D-23-00080-supplement.pdf

## Notes

**Peer Reviewed**

**First published online:** September 25, 2023.
