# Supplementary material for: Strengthening Integrated Approaches for Family Planning and Menstrual Health
Source: Glob Health Sci Pract. 2023 Oct 30;11(5):e2300080. doi: 10.9745/GHSP-D-23-00080 (PMC10615238; doi:10.9745/GHSP-D-23-00080)
Supplement: GHSP-D-23-00080-supplement.pdf [file GHSP-D-23-00080-supplement.pdf]

**Supplement to**: Hoppes E, Rademacher KH, Wilson L, et al. Strengthening integrated approaches for family planning and menstrual health. *Glob Health Sci Pract*. 2023;11(5):e2300080.
https://doi.org/10.9745/GHSP-D-23-00080

<table>
<tr><th colspan="5">Programmatic Guidance for Family Planning and Menstrual Health Integration Across Important Stages in the Reproductive Life Course</th></tr>
<tr><th></th><th>Preadolescence</th><th>Adolescence</th><th>Mid-Life and Reproductive Years</th><th>Perimenopause and Menopause</th></tr>
<tr><td rowspan="4">Improve Education and Awareness</td><td colspan="2">Include evidence-based puberty and comprehensive sexuality education for youth/adolescents that includes age-appropriate information on menstruation and fertility, managing menstrual bleeding and pain, menstrual health, and family planning , including contraceptive-induced menstrual changes, across settings, including for both in and out of school youth of all genders. Follow evidence-informed guidance on comprehensive sexuality education.9</td><td>Continue to provide evidence-based sexuality education and information about menstruation and fertility, managing menstrual bleeding and pain, menstrual health, and family planning, including contraceptive-induced menstrual changes, during this time.</td><td>Continue to provide evidence-based sexuality education and information about menstruation and fertility, managing menstrual bleeding and pain, menstrual health, and family planning, as well as information on perimenopause and menopause.</td></tr>
<tr><td colspan="4">Expanding access to evidence-based tools that provide access to family planning and menstrual health education, products, and services to people of all ages and genders, including tools that can be accessed directly by individuals through digital channels. Examples of existing tools that provide integrated information include AskNivi,[10] Managing Menstruation: Know Your Options,[11] Natural Cycles[12] and Love Matters.[13]</td></tr>
<tr><td colspan="2">Train and strengthen the capacity of community health workers and hold them accountable in providing age-appropriate, sensitive, youth-responsive, evidence-based counseling and education on menstruation and fertility, managing menstrual bleeding, pain, and disorders, menstrual health, and family planning, including contraceptive-induced menstrual changes.</td><td>Train and strengthen the capacity of community health workers and hold them accountable in providing sensitive, evidence-based counseling and education about menstruation and fertility, managing menstrual bleeding pain, and disorders, menstrual health, and family planning, including contraceptive-induced menstrual changes and post-partum return to fertility.</td><td>Train and strengthen the capacity of community health workers and hold them accountable in providing sensitive evidence-based counseling and education about menstruation and fertility, managing menstrual bleeding, pain and disorders, menstrual health, and family planning, including contraceptive-induced menstrual changes, as well as information on perimenopause and menopause.</td></tr>
<tr><td colspan="2">Provide support information and education to stakeholders such as educators and school staff, parents/guardians, community-based workers (across sectors), peer educators, and community and faith leaders that aligns with and supports the education provided by schools, community health workers, and other channels.</td><td>Train and strengthen the capacity of stakeholders such as community-based workers (across sectors) and community and faith leaders to provide sensitive evidence-based education about family planning, menstrual health, and contraceptive-induced menstrual changes.</td><td>Train and strengthen the capacity of stakeholders such as community-based workers (across sectors) and community and faith leaders to provide sensitive evidence-based education about family planning, menstrual health, and contraceptive-induced menstrual changes throughout perimenopause and until menopause is confirmed.</td></tr>
<tr><td rowspan="5">Improve Systems-Level Interactions</td><td></td><td colspan="3">Integrate delivery of menstrual health commodities and services into family planning within health systems. Provide affordable, high-quality menstrual health products, facilities, including clean, private toilets with space for washing and disposal, and other resources to family planning clients during counseling and/or service provision and/or referrals for products and services. Recognize that family planning users may need more, less, or different menstrual health products when they are using contraception and that this can change over time.</td></tr>
<tr><td></td><td colspan="3">Integrate the delivery of family planning commodities and services into menstrual health programs. Provide affordable, high-quality family planning services, including counseling and method provision and/or referrals for family planning services as part of both school and community-based menstrual health programs.</td></tr>
<tr><td></td><td colspan="3">Train and strengthen the capacity of providers and hold them accountable to delivering integrated care. Ensure that those providing sexual and reproductive health services are trained in both comprehensive family planning and menstrual health counseling as described in the section above, including training on contraceptive-induced menstrual changes and management of menstrual disorders, as well as youth-responsive services.</td></tr>
<tr><td></td><td colspan="3">Promote self-care,[29] including self-reassurance about contraceptive-induced menstrual changes. Ensure people have the information they need and reliable access to menstrual health products, facilities, and other resources including self-care options for menstrual pain. Support individuals to gain the self-efficacy and bodily autonomy they need to use resources with confidence.</td></tr>
<tr><td colspan="4">Ensure family planning users are included in menstrual health research and programs, including research involving menstrual products and development of menstrual health standards. Consider the needs and preferences of end-users in a holistic way that explicitly includes both family planning and menstrual health when designing and implementing menstrual health or family planning research, programs, products, systems, and standards.</td></tr>
</table>

**Supplement to**: Hoppes E, Rademacher KH, Wilson L, et al. Strengthening integrated approaches for family planning and menstrual health. *Glob Health Sci Pract*. 2023;11(5):e2300080.
https://doi.org/10.9745/GHSP-D-23-00080

<table>
<tr><th colspan="5">Programmatic Guidance for Family Planning and Menstrual Health Integration Across Important Stages in the Reproductive Life Course</th></tr>
<tr><th></th><th>Preadolescence</th><th>Adolescence</th><th>Mid-Life and Reproductive Years</th><th>Perimenopause and Menopause</th></tr>
<tr><td></td><td colspan="4">Revise health management information systems and reporting tools to support and report on integrated care, including adding information about menstrual health to existing family planning registers.</td></tr>
<tr><td rowspan="8">Improve Client-Level Interactions within Health Systems</td><td colspan="4">Provide information and counseling on menstrual health, including information about the full range of available options for managing menstrual (and contraceptive-induced) bleeding and pain, including information on self-care options and if feasible, access to, or at least information on where to access, commercial menstrual products locally, using tools such as Managing Menstruation: Know Your Options. Recognize that family planning users may need more, less, or different menstrual health products when they are using contraception and that this can change over time.[11]</td></tr>
<tr><td colspan="2">Provide access to comprehensive youth-responsive services[30] that include menstrual health education and information about the full range of menstrual health and family planning, including self-care options, to ensure smooth transition into puberty and to ensure that future menstrual health and family planning needs are met as soon as they arise. Ensure that services are age-appropriate and welcome all genders.</td><td colspan="2"></td></tr>
<tr><td></td><td colspan="3">Provide information on family planning, including on the full contraceptive method mix including complete and correct information about fertility-based awareness methods and lactation amenorrhea method options.[a] If the client chooses to use family planning, provide effective, evidence-based counseling during and after method selection about potential contraceptive-induced menstrual changes, using provider job aids such as the NORMAL tool.[34]</td></tr>
<tr><td></td><td colspan="3">Provide adequate support and clinical treatment for undesirable contraceptive-induced menstrual changes. Ensure adequate follow-up services and counsel family planning users that they can return at any time if they have questions or concerns.</td></tr>
<tr><td></td><td colspan="2">Provide effective, evidence-based post-partum counseling including products for the management of postpartum bleeding, information about the return of menstruation and fertility after pregnancy and after family planning use, as well as support on tapering off family planning when trying to conceive.</td><td></td></tr>
<tr><td></td><td colspan="3">Promote use of the pregnancy checklist[41] and/or provide access to low-cost pregnancy tests to ensure same-day provision of contraceptive methods among women seeking services when they are not menstruating (i.e., to ensure providers do not rely on the presence of menses as an indicator that a client is not pregnant before providing contraceptive methods.)</td></tr>
<tr><td colspan="3"></td><td>Provide information and services related to perimenopause and menopause, including how to manage symptoms and counsel on contraceptive use during this life stage. Also ensure perimenopausal people have access to low-dose contraceptives as an option to relieve menopause symptoms.</td></tr>
<tr><td colspan="4">Ask about and address concerns about menstruation and menstrual discomfort, including diagnosis and treatment of disorders. Even in the absence of a diagnosis, provide information on management of symptoms should include education on self-care (light exercise, stretching and/or yoga, applying heat such as a hot water bottle, taking ibuprofen or naproxen, and other evidence-based self-care options), contraception, and other available options.</td></tr>
<tr><td rowspan="2">Reach Populations with Special Needs</td><td></td><td colspan="3">When providing services to populations with special or unique needs[b], ensure that both their menstrual health and family planning needs are adequately addressed and counsel on contraceptive-induced menstrual changes accordingly. Ensure that individuals are not denied their rights to information about sexual and reproductive health, including menstrual health and family planning and consent to family planning method use/provision.</td></tr>
<tr><td></td><td colspan="3">Ensure populations with special or unique needs are included in family planning and menstrual health research and product introduction programs and that these research studies and programs are informed by the populations they are serving and designed to be as accessible as possible.</td></tr>
</table>

**Supplement to**: Hoppes E, Rademacher KH, Wilson L, et al. Strengthening integrated approaches for family planning and menstrual health. *Glob Health Sci Pract*. 2023;11(5):e2300080.
https://doi.org/10.9745/GHSP-D-23-00080

| Programmatic Guidance for Family Planning and Menstrual Health Integration Across Important Stages in the Reproductive Life Course | | | | |
|---|---|---|---|---|
| | Preadolescence | Adolescence | Mid-Life and Reproductive Years | Perimenopause and Menopause |
| | | **Ensure all individuals with menstrual discomfort and /or disorders** have adequate counseling and access to contraception as a management or prevention option. | | |
| | | **Ensure people with menstrual disorders are included in** family planning **and** menstrual health **research and product introduction programs when possible.** | | |
| **Implement Advocacy and SBCC Programs** | **Include messaging about** family planning**,** menstrual health**, and** contraceptive-induced menstrual changes **in social and behavior change communication campaigns and interventions,** including interventions that destigmatize and make these topics more understandable to relevant audiences. | | | |
| | **Advocate at the policy level** to ensure decision-makers are educated on the issues of family planning, menstrual health, and contraceptive-induced menstrual changes and aware of the best ways to include these issues in policy-level decisions. | | | |
| **Strengthen National Policies and Guidelines** | **Review and update** family planning**,** menstrual health**, and sexual and reproductive health and rights policies** to ensure that adequate, evidence-based information about family planning - menstrual health integration and contraceptive-induced menstrual changes is included and promoted | | | |
| | **Review and update** family planning**,** menstrual health**, and sexual and reproductive health and rights guidelines** to ensure that adequate, evidence-based information about family planning - menstrual health integration and contraceptive-induced menstrual changes is included. Update clinical guidance and training for healthcare providers, as needed. | | | |
| In all areas of integration, **conduct programmatic research, implementation science, and routine or enhanced monitoring and evaluation** that can be used to inform and improve future programs.[3] | | | | |
